# The current situation and influencing factors of occupational grief among clinical nurses: A scoping review

**DOI:** 10.1097/MD.0000000000046468

**Published:** 2025-12-19

**Authors:** Chenxi Yin, Xiaoshan Chen, Yong Kou, Zhongqing Yuan

**Affiliations:** a Department of Abdominal Tumor, West China Hospital, Sichuan University, Chengdu, Sichuan, China; b Department of Respiratory and Critical Care Medicine, West China Hospital, Sichuan University, Chengdu, Sichuan, China; c Sichuan Nursing Vocational College, Chengdu, Sichuan, China.

**Keywords:** clinical nurses, nursing mental health, occupational grief, scoping review

## Abstract

**Background:**

Occupational grief, a psychological response arising from repeated exposure to patient suffering and death, is increasingly recognized among clinical nurses. This emotional burden not only impairs mental health but also affects professional functioning and patient care quality. Despite its relevance, there has been no comprehensive synthesis of the tools used to assess occupational grief, its contributing factors, and potential interventions.

**Methods:**

This scoping review followed the Arksey and O’Malley framework and adhered to Preferred Reporting Items for Systematic Reviews and Meta-Analyses Extension for Scoping Reviews guidelines. We systematically searched 9 English and Chinese databases – including PubMed, Web of Science, Embase, Cochrane Library, China National Knowledge Infrastructure, and others – up to December 30, 2024. Studies were included if they assessed occupational grief among clinical nurses using validated tools and reported influencing factors. Two reviewers independently screened studies and extracted data. Descriptive synthesis was conducted on measurement tools, associated factors, intervention strategies, and related health outcomes.

**Results:**

A total of 14 cross-sectional studies were included, encompassing over 4000 clinical nurses from diverse departments such as intensive care units, oncology, and emergency care. The revised grief experience inventory, Pandemic Grief Scale, and Death Coping Self-Efficacy Scale were among the most frequently used tools. Occupational grief was found to be significantly associated with psychological factors, professional characteristics, and environmental or organizational elements. Proposed interventions included psychological detachment training, grief education, organizational support, and structured peer support programs.

**Conclusion:**

Occupational grief is prevalent among clinical nurses and is shaped by a complex interplay of personal, professional, and organizational factors. Standardized tools like The revised grief experience inventory and Death Coping Self-Efficacy Scale have enabled more consistent assessment, but further longitudinal and interventional studies are warranted. Enhancing nurses’ coping capacity through education, support systems, and workplace reforms may help mitigate the negative impact of occupational grief.

## 1. Introduction

Nurses are frequently exposed to emotionally charged situations, particularly in clinical settings involving critical care, palliative care, and end-of-life decision-making. Among the various emotional responses arising from these experiences, occupational grief has emerged as an important yet relatively underexplored phenomenon. occupational grief refers to the emotional distress experienced by healthcare providers due to patient death or deterioration during clinical practice.^[1]^ This form of distress typically manifests through symptoms such as profound sadness, self-blame, insomnia, appetite loss, and intrusive thoughts related to death.^[2]^ Due to the unique nature of the caregiver–patient relationship, the grief experienced by healthcare professionals differs from familial bereavement and is often categorized as disenfranchised grief – grief that is not openly acknowledged or socially validated.^[3]^

Existing research emphasizes that healthcare workers are at high risk for occupational burnout, with studies indicating that between 37% and 53% of healthcare providers experience symptoms such as emotional exhaustion, depersonalization, and reduced personal accomplishment.^[4]^ Foundational scholars such as Maslach and Leiter have identified critical organizational factors – such as workload, autonomy, rewards, team cohesion, value congruence, and perceptions of fairness – as key drivers of burnout.^[5]^ Although occupational burnout and occupational grief are distinct concepts, they often coexist in emotionally demanding clinical environments. When experiencing occupational grief, many healthcare professionals adopt maladaptive coping mechanisms, including avoidance of death-related environments^[6,7]^ or even substance misuse as a way to manage grief-related emotional distress.^[8]^

Despite increasing recognition of occupational grief, the literature remains fragmented across different clinical specialties, geographical settings, and research methodologies. A comprehensive understanding of the coping mechanisms employed by medical professionals in response to occupational grief remains lacking.

Therefore, this scoping review aims to systematically synthesize the existing evidence on the definition, assessment tools, influencing factors, and coping strategies related to nurses’ occupational grief. In addition, this review seeks to identify gaps in the current literature and suggest directions for future research to enhance the mental health and professional sustainability of clinical nursing staff.

## 2. Methods

### 2.1. Design

This scope review followed the methodological framework proposed by Arksey and O’Malley (2005), was improved by Levac et al,^[9,10]^ and was reported based on the Preferred Reporting Items for Meta-Analyses Extended by Systematic Review and Scope Review list. The 5-stage process includes: determining the research question, identifying relevant research, selecting eligible research, plotting data charts, and organizing, summarizing and reporting the results.

### 2.2. Qualification criteria

The inclusion and exclusion criteria were included if they met the following criteria: published in Chinese or English before December 30, 2024; participants were registered nurses with at least 6 months of frontline clinical work experience; occupational grief was assessed using standardized scales; and analysis of influencing factors for occupational grief was conducted. A control group was not required for inclusion.

Studies were excluded if they: involved non-adult populations; were duplicate publications; were qualitative studies, reviews, meta-analyses, commentaries, or opinion pieces without empirical data; or did not provide full-text access.

Although the inclusion criteria required participants to have at least 6 months of frontline clinical experience, most studies included in this review involved nurses with 3 to 10 years of clinical practice, with an average age between 30 and 34 years.

### 2.3. Information sources

A systematic search was conducted across 9 electronic databases: PubMed, Web of Science, EMBASE, The Cochrane Library, CINAHL, China National Knowledge Infrastructure, Wanfang Data, VIP Database, and the China Biomedical Literature Database. Detailed search strategies are provided in File S1, Supplemental Digital Content, https://links.lww.com/MD/Q864. Additional eligible studies were identified by manually screening the reference lists of included articles.

This review was structured around the following research questions:

1. What standardized tools or scales are used to assess occupational grief among clinical nurses?
2. What individual, occupational, or organizational factors are associated with the presence or severity of occupational grief?
3. What intervention measures have been proposed to reduce occupational grief?
4. What health outcomes are related to occupational grief?

### 2.4. Data extraction and management

Two reviewers (CXY and XSC) independently extracted data using a standardized form, capturing information on study design, methodology, participant characteristics, assessment tools, influencing factors, interventions, and health outcomes. Discrepancies were resolved through discussion or consultation with a third reviewer (YK) when necessary. If critical data were missing, corresponding authors were contacted to obtain further information. All extracted data were managed using Excel spreadsheets. The study selection process was documented using a PRISMA-compliant flowchart.^[11]^

### 2.5. Bias risk (quality) assessment

Although quality assessment is not mandatory for scoping reviews, it was incorporated to enhance the credibility of our findings, in line with recommendations by Levac et al and Daudt et al.^[10,12]^ Two independent reviewers (CXY and XSC) critically appraised the included studies using the National Heart, Lung, and Blood Institute quality assessment tools for observational cohort and cross-sectional studies, as well as case series studies.^[13]^ Studies were rated as “good,” “fair,” or “poor” quality based on predefined criteria. Studies assessed as “poor” quality were excluded from the final synthesis but are presented in Supplementary.

### 2.6. Data charting and synthesis

Key data were charted and synthesized in tabular form according to the review objectives. Information extracted included authorship, country of origin, study design, sample size, study topic, assessment tools, influencing factors, intervention strategies, and reported health outcomes. A narrative summary accompanied the tabulated results.

## 3. Result

### 3.1. Study selection and characteristics

**Figure 1.**
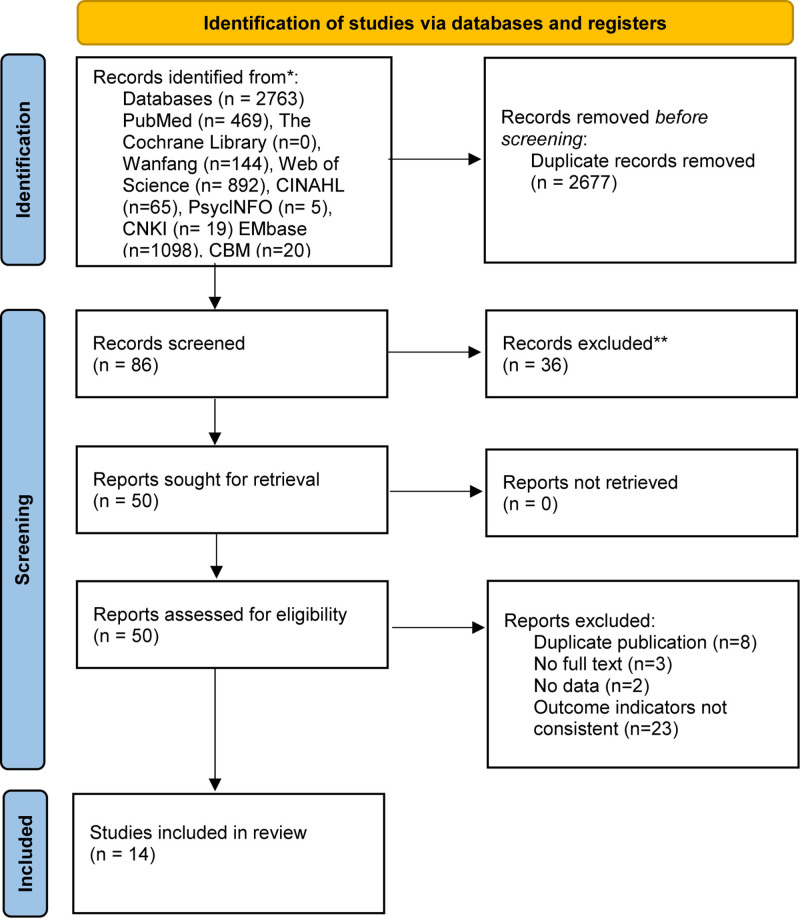
PRISMA 2020 flow diagram of the study selection process. The figure illustrates the identification, screening, eligibility assessment, and final inclusion of studies in the systematic review and meta-analysis. A total of 2763 records were initially identified from various databases. After removing duplicates, 86 records were screened, resulting in the inclusion of 14 studies. CNKI = China National Knowledge Infrastructure.

A total of 2763 records were retrieved from electronic databases, including PubMed (n = 469), Wanfang (n = 144), Web of Science (n = 892), CINAHL (n = 65), PsycINFO (n = 5), China National Knowledge Infrastructure (n = 19), Embase (n = 1098), and China Biomedical Literature Database (n = 20). After removing 2677 duplicate records, 86 records remained for screening. Following the title and abstract screening, 36 records were excluded, and 50 full-text articles were assessed for eligibility. Of these, 36 were excluded for the following reasons: duplicate publication (n = 8), no full text available (n = 3), missing data (n = 2), or inconsistent outcome indicators (n = 23). Finally, 14 cross-sectional studies were included in this scoping review (Fig. 1). The basic characteristics of these studies are summarized in Table 1.

**Table 1:** Basic characteristics of the included literature.

References	Type of study	Sample size (Male/Female)	Sampling method	Sampling method	Sampling method (Yr)	Department	Country	Assessment tool	Occupational Grief Score	Standardized Occupational Grief Score	Sub-dimensions reported	Sub-dimensions reported	NHLBI score
Zuo et al^[14]^	Cross-sectional	199 (3/196)	Convenience sampling	34	3	Palliative Care	China	RGEI	37.00 (27.00,50.00)	0.54	Yes	①⑤⑦⑧⑪	Fair
Wan et al^[15]^	Cross-sectional	366 (61/305)	Convenience sampling	NR	≥1.00	Emergency Department	China	RGEI	44.60 ± 5.05	0.81	Yes	①②③④⑥⑤⑧⑨⑪㉙	Fair
Xiong et al^[16]^	Cross-sectional	241 (27/214)	Convenience sampling	NR	>1.00	General Ward	China	RGEI	43.54 ± 5.41	0.79	Yes	①⑧⑬㉙	Good
Gao et al^[17]^	Cross-sectional	308 (24/282)	Convenience sampling	NR	≥1.00	ICU	China	RGEI	36.00 ± 7.01	0.50	No	⑬⑭⑮⑲⑳	Fair
Xu et al^[18]^	Cross-sectional	462 (69/393)	Convenience sampling	31.4	>10.00	Oncology Department	China	Sadness State Scale	51.72 ± 7.64	0.51	Yes	①⑤⑩⑪㉕	Fair
Long et al^[19]^	Cross-sectional	262 (60/202)	Convenience sampling	NR	>10	ICU	China	PBS-AGC	55.02 ± 8.03	0.67	Yes	⑯⑪㉙	Fair
Zhang et al^[20]^	Cross-sectional	234	Convenience sampling	31.46 ± 7.60	NR	Palliative Care	China	RGEI	37.49 ± 3.83	0.55	Yes	⑩㉕⑰	Fair
Kim et al^[21]^	Cross-sectional	229 (13/216)	Convenience sampling	30.10 ± 6.30	6.90 ± 6.00	COVID-19 Ward	South Korea	PGS	1.40 ± 2.90	0.09	Yes	⑲⑳㉒	Good
Lin et al^[22]^	Cross-sectional	572 (3/569)	Convenience sampling	32.40 ± 7.10	<10 yr account for 73.1%	Surgery, Internal Medicine, Emergency, etc	China	DCSS	102.58 ± 12.07	0.50	Yes	④㉓㉘	Good
Rahmani et al^[23]^	Cross-sectional	375 (137/238)	Convenience sampling	31.90 ± 8.30	8.34 ± 3.50	COVID-19 Ward	Iran	ICG	29.40 ± 7.10	0.39	Yes	⑨⑫⑱㉗	Good
Gong et al^[24]^	Cross-sectional	620 (8/612)	Convenience sampling	32.07 ± 5.32	5.16 ± 3.00	Oncology Department	China	DCSS	133.57 ± 26.78	0.57	Yes	㉘⑥㉔⑬	Good
Turgut and Yildiz^[25]^	Cross-sectional	200 (36/164)	Convenience sampling	32.84 ± 7.69	3.75 ± 2.05	ICU	Turkey	TRIG	49.425 ± 10.86	0.71	Yes	㉓㉔⑬	Good
Park et al^[26]^	Cross-sectional	267 (57/210)	Convenience sampling	31.40 ± 5.40	6.60 ± 5.40	COVID-19 Ward	South Korea	PGS	4.70 ± 4.40	0.16	Yes	㉖⑳⑲	Good
Hong et al^[27]^	Cross-sectional	251 (57/194)	Convenience sampling	29.40 ± 6.20	6.40 ± 5.70	COVID-19 Ward	South Korea	PGS	2.60 ± 3.50	0.17	Yes	⑲㉑㉒	Good

This table summarizes key information of each included study, including study type, sample size, setting, country, assessment tools, and standardized occupational grief scores. ① Religious belief; ② Type of exposure to death; ③ Frequency of exposure to death; ④ Hospice care education ⑤ Hospice care attitude; ⑥ Death education; ⑦ Anxiety about death; ⑧ Psychological detachment; ⑨ Support society; ⑩ For professional title; ⑪ Represents age; ⑫ Gender; ⑬ Length of service; ⑭ Perceived decent work; ⑮ Occupational well-being; ⑯ Sense of meaning in life; ⑰ Emotional labor; ⑱ Ward type (e.g., ICU, COVID-19 ward); ⑲ Depression; ⑳ Anxiety; ㉑ Loneliness; ㉒ Fear of viruses; ㉓ Bereavement experience; ㉔ Comfort in providing support; ㉕ Sadness state; ㉖ Work pressure; ㉗ Shift type; ㉘ Attitude toward death ㉙ Years of service.

DCSS = Death Coping Self-Efficacy Scale, ICG = inventory of complicated grief, NHLBI = National Heart, Lung, and Blood Institute, NR: unreported, PBS-AGC = Professional Bereavement Scale for Acute Grief in Caregivers, PGS = Pandemic Grief Scale, RGEI = Revised Grief Experience Inventory, TRIG = Texas Revised Inventory of Grief.

All 14 included studies used cross-sectional designs, with sample sizes ranging from 199 to 620 participants.^[14–27]^ Convenience sampling was employed in all studies. The participants were clinical nurses from various departments, including palliative care, emergency, intensive care units (ICU), oncology, and COVID-19-specific wards. Most studies were conducted in China (n = 10), followed by South Korea (n = 3), Iran (n = 1), and Turkey (n = 1). Participants were predominantly female (over 90%), with an average age between 30 and 34 years and work experience ranging from 3 to 10 years.

The revised grief experience inventory (RGEI) was the most frequently used instrument to assess occupational grief, followed by Professional Bereavement Scale for acute grief in caregivers, Death Coping Self-Efficacy Scale (DCSS), inventory of complicated grief, Texas revised inventory of grief, and Pandemic Grief Scale (PGS). Thirteen studies reported sub-dimensional results related to occupational grief, typically involving stress, guilt, depressive symptoms, and grief-related emotional states. Standardized occupational grief scores ranged from 0.09 to 0.81, indicating notable variability in grief experiences among nurses across different settings.

### 3.2. Measurement methods for occupational grief

Six different instruments were used to assess occupational grief among clinical nurses in the included studies (Table 1). The most frequently adopted tool was the RGEI, which appeared in 6 studies.^[14–18,20]^ The RGEI includes 22 items across 4 dimensions: depressive mood, stress, guilt, and physical symptoms. Standardized scores derived from RGEI ranged from 0.50 to 0.81, suggesting that most participants experienced moderate levels of occupational grief.

Other measurement tools included the Professional Bereavement Scale for acute grief in caregivers,^[19]^ the DCSS,^[22,24]^ the inventory of complicated grief,^[23]^ the Texas revised inventory of grief,^[25]^ and the PGS.^[21,26,27]^ Notably, the PGS was specifically developed for use in the context of the COVID-19 pandemic and is designed for rapid, large-scale screening of pandemic-related grief symptoms.

### 3.3. Factors related to occupational grief

This scoping review identified multiple factors associated with occupational grief among clinical nurses, which can be categorized into 3 domains: personal, occupation-related, and environmental or supportive factors (see Table 2). Personal factors were reported in 8 studies^[14–21]^ and included religious beliefs, perceived meaning in life, emotional labor intensity, and psychological symptoms such as depression, anxiety, and fear of death. Particularly during the COVID-19 pandemic, virus-related fear, loneliness, and emotional fatigue were highlighted as amplifiers of grief.^[21,27]^

**Table 2:** Factors associated with professional grief among clinical nurses in included studies.

References	Personal factors	Occupation-related factors	Environmental or supporting factors
Zuo et al^[14]^	–	–	
Wan et al^[15]^	–	–	–
Xiong et al^[16]^	–	–	
Gao et al^[17]^	–	–	
Xu et al^[18]^	–	–	
Long et al^[19]^	–		
Zhang et al^[20]^	–		
Kim et al^[21]^	–		
Lin et al^[22]^		–	
Rahmani et al^[23]^		–	–
Gong et al^[24]^		–	
Turgut and Yildiz^[25]^		–	
Park et al^[26]^			–
Hong et al^[27]^			–

This table categorizes individual, occupational, and environmental factors identified as related to occupational grief.

Occupation-related factors were discussed in 9 studies,^[15–18,22–25]^ including years of service, professional title, department type (e.g., ICU, oncology, and COVID-19 wards), frequency of death exposure, and rotating shift patterns. Nurses working in high-intensity environments were more likely to experience elevated levels of grief. Environmental or supportive factors appeared in 5 studies,^[15,23–27]^ emphasizing the protective role of peer support, organizational recognition of grief, and access to psychological services. Across these studies, occupational grief was frequently associated with negative psychological outcomes, including depression, anxiety, and compassion fatigue.

### 3.4. Health outcomes related to occupational grief

Occupational grief among clinical nurses exerts multidimensional effects on mental health, occupational functioning, and potential for positive psychological growth (see Table 3). A total of 13 studies^[14–26]^ reported significant associations between occupational grief and adverse mental health outcomes, such as depression, anxiety, stress, and emotional exhaustion. Particularly during the COVID-19 pandemic, symptoms including loneliness, insomnia, and intensified anxiety were prevalent,^[22,27]^ suggesting that grief not only increases emotional burden but also diminishes nurses’ psychological resilience.

**Table 3:** Health outcomes related to occupational grief.

Health outcome category	Specific outcomes	References
Mental health impacts	Depression, anxiety, stress, death anxiety, emotional exhaustion, loneliness, and insomnia	[14–16,18,21,22,24–27]
Occupational function impacts	Burnout, compassion fatigue, turnover intention, reduced job satisfaction, and impaired empathy	[16–18,20,23–25]
Positive psychological growth	Reduced sense of meaning in life and restricted post-traumatic growth (PTG)	[19,25]

This table lists the mental health, occupational function, and positive psychological outcomes associated with occupational grief reported in the included studies.

Regarding occupational functioning, 10 studies^[16–18,20,23–25]^ indicated that grief was associated with burnout, compassion fatigue, decreased job satisfaction, and reduced empathy factors that negatively impact clinical performance and elevate turnover intention. Evidence on positive psychological growth was relatively limited. One study^[19]^ reported a decline in perceived meaning in life, while another^[25]^ noted constrained potential for post-traumatic growth in nurses experiencing persistent grief.

### 3.5. Intervention measures related to occupational grief

Intervention strategies aimed at mitigating occupational grief among clinical nurses can be broadly classified into 3 domains: organizational management interventions, psychological and emotional support interventions, and educational and capacity-building interventions (see Table 4). A total of 11 studies^[14,15,17,19,20,23–27]^ recommended organizational approaches, which included flexible shift scheduling,^[14,15,23,26]^ job rotation mechanisms,^[23]^ institutional or preservice training programs,^[14,24]^ improvements in salary and welfare benefits,^[17,19]^ and promotion of supportive organizational culture.^[20]^ Additionally, structural strategies such as risk identification mechanisms,^[26]^ recovery time allocation,^[25]^ and systematic evaluation protocols^[27]^ were proposed to safeguard mental well-being.

**Table 4:** Intervention categories and strategies for occupational grief.

Intervention category	Specific strategies	References
Organizational management interventions	Flexible shift scheduling	[14,15,23,26]
	Job rotation mechanism	[23]
	Preservice or institutional training arrangements	[14,24]
	Salary and benefits improvement	[17,19]
	Organizational culture building	[20]
	Risk identification mechanism	[26]
	Recovery time mechanisms	[25]
	Systematic evaluation strategies	[27]
Psychological and emotional support interventions	Mindfulness or emotional regulation training	[14]
	Psychological detachment strategies	[15,16]
	Social support mechanisms	[15]
	Grief counseling	[23]
	Emotional expression training	[20]
	Positive death attitude guidance	[22]
	Psychological release or resilience building	[18,27]
	Organizational psychological support	[14,19,21]
	Religious or spiritual support	[25]
	Early psychological intervention strategies	[26]
Educational and capacity enhancement interventions	Continuing education and general training	[14,19]
	Death education and hospice care training	[15,18,22]
	Emotional labor or humanities courses	[20]
	Development of grief or coping assessment tools	[21]
	Construction of identification mechanisms	[23]
	Personalized death education	[24]
	End-of-life care training	[25]
	Intervention based on assessment scales	[26]
	Measurement-based intervention proposals	[27]

This table summarizes organizational management, psychological support, and educational interventions proposed to address occupational grief among clinical nurses.

Psychological and emotional support interventions were discussed in 12 studies,^[14–16,18–23,25–27]^ focusing on strengthening emotional resilience through mindfulness or emotion regulation training,^[14]^ psychological detachment techniques,^[15,16]^ grief counseling,^[23]^ and emotional expression facilitation.^[20]^ Several studies also emphasized the value of religious or spiritual support,^[25]^ organizational psychological support systems,^[14,19,21]^ peer and institutional social support,^[15]^ and early psychological intervention mechanisms.^[26]^

Educational and capacity-building interventions were the most frequently cited, appearing in 13 studies.^[14,15,18–20,22–27]^ These interventions encompassed continuing education programs and general skills training,^[14,19]^ death education and hospice care training,^[15,18,22]^ emotional labor and humanities curriculum,^[20]^ personalized grief-related education,^[24]^ and the development of assessment tools or scale-based intervention frameworks.^[21,23,26,27]^ Collectively, these strategies aim to enhance nurses’ preparedness and competence in addressing death-related challenges and managing grief constructively.

## 4. Discussion

### 4.1. Summary of evidence

This scoping review confirms that occupational grief is prevalent among clinical nurses and has multidimensional impacts on their mental health, professional functioning, and organizational outcomes. Studies consistently show that frequent exposure to patient death, high-pressure environments, and intense emotional labor substantially contribute to increased levels of occupational grief. These findings align with those of Chang et al,^[28]^ who highlighted grief as a common psychological response in end-of-life care settings characterized by recurrent loss and emotional strain.

Cross-regional comparisons revealed that nurses in China and the Middle East reported higher levels of occupational grief compared to those in South Korea,^[29]^ potentially attributable to shortages in nursing resources, heavy workloads, lack of systematic emotional support mechanisms, and cultural differences in attitudes toward death. Occupational grief has been shown to correlate positively with depression, anxiety, emotional exhaustion, and burnout,^[30,31]^ and is linked to impaired occupational functioning, including compassion fatigue, turnover intention, and reduced job satisfaction. According to the Job Demands-Resources (JD-R) model, the combination of high job demands, frequent death encounters, emotional labor burden, and limited personal resources (e.g., low resilience and insufficient social support) exacerbates burnout and emotional exhaustion, further leading to deteriorated care quality and weakened professional identity^[32,33]^

In this review, social support systems, psychological detachment strategies, and positive death education are regarded as buffer factors to alleviate the adverse effects of occupational grief. Consistent with previous research results, higher work resources are associated with reduced stress, enhanced motivation, and positive health outcomes among caregivers.^[34,35]^ Furthermore, a qualitative study exploring the working conditions of surgical specialist nurses in Sweden indicates that the job resources in the working environment can affect engagement, enthusiasm, happiness, as well as opportunities for career growth and development.^[36]^

This review also identified that occupational grief is influenced by an interplay of personal, occupational, and environmental factors. Specifically, nurses with more than 10 years of clinical experience exhibited higher grief scores compared to their less experienced counterparts, ICU and emergency nurses also reported significantly greater occupational grief than nurses in other departments, likely due to the severity of patient conditions and high mortality rates encountered. These findings are consistent with Chen et al integrated model of occupational grief, which emphasizes the cumulative impact of repeated patient deaths on healthcare professionals’ personal and professional domains.^[37]^

This study finds, structured assessment tools: RGEI, DCSS, generally yielded higher grief scores than brief instruments such as the PGS, suggesting that tool selection critically influences outcome estimates. Other factors, including religious beliefs, hospice education experience, psychological detachment capacity, and perceived comfort in providing support, were also significantly associated with occupational grief levels. However, religious belief was less frequently examined in Chinese studies, likely reflecting a relatively secular societal context compared to Western countries.^[38]^

Overall, these findings highlight that occupational grief is shaped not only by environmental factors but also by individual cognitive appraisals and emotional coping strategies.

### 4.2. Advantages and limitations

Several strengths and limitations should be noted in this review. A major strength is the rigorous implementation of a recognized scoping review framework, supplemented by Preferred Reporting Items for Systematic Reviews and Meta-Analyses Extension for Scoping Reviews reporting guidelines, comprehensive database searching, and independent screening and data extraction by 2 reviewers. Quality appraisal was conducted using the National Heart, Lung, and Blood Institute tool, enhancing the credibility of the findings.

However, several limitations exist. First, the included studies were predominantly cross-sectional, limiting causal inferences. Second, most samples were derived from single centers or specific departments, reducing the generalizability of results. Third, substantial heterogeneity in measurement tools across studies impeded direct comparisons and synthesis. Fourth, relatively few studies addressed intervention strategies, and most interventions were not formally tested for efficacy.

## 5. Conclusion

This scoping review systematically summarized the current status, influencing factors, intervention measures, and associated health outcomes related to occupational grief among clinical nurses. Occupational grief is highly prevalent and has profound impacts on nurses’ mental well-being, professional performance, and growth potential. Future research should focus on the standardization of measurement tools, development of longitudinal and intervention studies, exploration of cross-cultural variations, and the establishment of localized grief management programs to better support the mental health and career sustainability of nursing professionals.

## 6. Implications for nursing management

The widespread existence of occupational grief suggests that nursing managers need to attach great importance to the protection of nurses’ mental health. It is suggested to start from the following aspects: First, optimize organizational management, such as reasonable shift scheduling, establishing psychological support channels, and creating a mutual assistance cultural atmosphere. Second, strengthen vocational education and training to enhance nurses’ professional capabilities in dealing with death and grief. Third, establish a systematic sadness screening and intervention mechanism to identify high-risk groups at an early stage and intervene in a timely manner. Fourth, promote positive psychological growth and enhance the psychological resilience and professional identity of the nurse group. Through comprehensive and multi-level intervention strategies, the negative impact of occupational grief can be effectively mitigated, and the job satisfaction and sustainable career development ability of nursing staff can be improved.

Supplemental Digital Contents “File S2 and Figure S1” are available for this article (https://links.lww.com/MD/Q864; https://links.lww.com/MD/Q865).

## Author contributions

**Conceptualization:** Zhongqing Yuan.

**Data curation:** Zhongqing Yuan.

**Funding acquisition:** Zhongqing Yuan.

**Investigation:** Chenxi Yin.

**Methodology:** Chenxi Yin, Xiaoshan Chen, Zhongqing Yuan.

**Project administration:** Chenxi Yin.

**Resources:** Yong Kou, Zhongqing Yuan.

**Software:** Xiaoshan Chen, Yong Kou.

**Supervision:** Yong Kou.

**Writing – original draft:** Chenxi Yin.

**Writing – review & editing:** Chenxi Yin, Xiaoshan Chen, Yong Kou.

## Supplementary Material




