# Supplementary material for: The current situation and influencing factors of occupational grief among clinical nurses: A scoping review
Source: Medicine (Baltimore). 2025 Dec 19;104(51):e46468. doi: 10.1097/MD.0000000000046468 (PMC12727250; doi:10.1097/MD.0000000000046468)
Supplement: Supplementary file 1 [file medi-104-e46468-s001.docx]

Supplementary File 1: Search Strategy

PubMed

("nurses"[MeSH Terms] OR "clinical nurse" OR "registered nurse" OR "healthcare worker")

AND ("grief"[MeSH Terms] OR "professional grief" OR "occupational grief" OR "bereavement" OR "emotional burden")

AND ("prevalence" OR "incidence" OR "cross-sectional study" OR "occupational health")

Web of Science

TS = (("nurse*" OR "clinical nurse*" OR "registered nurse*")

AND ("grief" OR "professional grief" OR "emotional burden" OR "bereavement")

AND ("prevalence" OR "incidence" OR "status"))

**EMBASE**

('nurse'/exp OR 'clinical nurse' OR 'registered nurse')

AND ('grief'/exp OR 'occupational grief' OR 'professional grief' OR 'bereavement' OR 'emotional burden')

AND ('prevalence'/exp OR 'incidence' OR 'cross-sectional study' OR 'occupational health')

**CINAHL Search Strategy**

(nurse* OR "clinical nurse" OR "registered nurse")

AND ("grief" OR "occupational grief" OR "professional grief" OR "bereavement" OR "emotional burden")

AND ("prevalence" OR "incidence" OR "cross-sectional study")

**PsycINFO**

("nurse*" OR "clinical nurse*" OR "registered nurse*")

AND ("grief" OR "professional grief" OR "occupational grief" OR "bereavement" OR "emotional burden")

**CNKI /Wanfang / VIP / CBM**

(“护士” OR “临床护士” OR “注册护士”)

AND (“职业悲伤” OR “情绪负担” OR “哀伤” OR “哀伤反应”)

AND (“现状” OR “发生率” OR “影响因素”)

Supplementary File 2: Supplementary File 2. Quality Assessment of Included Studies Using the NHLBI Tool for Observational Cohort and Cross-Sectional Studies

| **Study** | **Q1** | **Q2** | **Q3** | **Q4** | **Q5** | **Q6** | **Q7** | **Q8** | **Q9** | **Q10** | **Q11** | **Q12** | **Q13** | **Q14** | **Total Score** | **Overall Quality** |
| --- | --- | --- | --- | --- | --- | --- | --- | --- | --- | --- | --- | --- | --- | --- | --- | --- |
| Zuo 2022 | Y | Y | N | Y | NR | Y | NR | NR | Y | N | Y | NR | Y | NR | 7/14 | Fair |
| Wan 2023 | Y | Y | N | Y | NR | Y | NR | NR | Y | N | Y | NR | Y | NR | 7/14 | Fair |
| Xiong 2023 | Y | Y | N | Y | Y | Y | Y | NR | Y | N | Y | NR | Y | NR | 9/14 | Good |
| Gao 2024 | Y | Y | N | Y | NR | Y | NR | NR | Y | N | Y | NR | Y | NR | 7/14 | Fair |
| Xu 2024 | Y | Y | N | Y | NR | Y | NR | NR | Y | N | Y | NR | Y | NR | 7/14 | Fair |
| Long 2023 | Y | Y | N | Y | NR | Y | NR | NR | Y | N | Y | NR | Y | NR | 7/14 | Fair |
| Zhang 2023 | Y | Y | N | Y | NR | Y | NR | NR | Y | N | Y | NR | Y | NR | 7/14 | Fair |
| Kim 2023 | Y | Y | Y | Y | NR | Y | NR | NR | Y | N | Y | NR | Y | NR | 8/14 | Good |
| Lin 2022 | Y | Y | Y | Y | NR | Y | NR | NR | Y | N | Y | NR | Y | NR | 8/14 | Good |
| Rahmani 2023 | Y | Y | Y | Y | NR | Y | NR | NR | Y | N | Y | NR | Y | NR | 8/14 | Good |
| Gong 2024 | Y | Y | Y | Y | NR | Y | NR | NR | Y | N | Y | NR | Y | NR | 8/14 | Good |
| Turgut 2023 | Y | Y | Y | Y | NR | Y | NR | NR | Y | N | Y | NR | Y | NR | 8/14 | Good |
| Park 2024 | Y | Y | Y | Y | NR | Y | NR | NR | Y | N | Y | NR | Y | NR | 8/14 | Good |
| Hong 2023 | Y | Y | Y | Y | NR | Y | NR | NR | Y | N | Y | NR | Y | NR | 8/14 | Good |

N: no; NA: not applicable; NHLBI: National Heart, Lung, and Blood Institute; RCT: randomized controlled trial; Y: yes.

Criteria：Q1. Was the research question or objective in this paper clearly stated? Q2. Was the study population clearly specified and defined? Q3. Was the participation rate of eligible persons at least 50%? Q4. Were all the subjects selected or recruited from the same or similar populations (including the same time period)? Were inclusion and exclusion criteria for being in the study prespecified and applied uniformly to all participants? Q5. Was a sample size justification, power description, or variance and effect estimates provided? Q6. For the analyses in this paper, were the exposure(s) of interest measured prior to the outcome(s) being measured? Q7. Was the timeframe sufficient so that one could reasonably expect to see an association between exposure and outcome if it existed? Q8. For exposures that can vary in amount or level, did the study examine different levels of the exposure as related to the outcome (e.g., categories of exposure, or exposure measured as continuous variable)? Q9. Were the exposure measures (independent variables) clearly defined, valid, reliable, and implemented consistently across all study participants? Q10. Was the exposure(s) assessed more than once over time? Q11. Were the outcome measures (dependent variables) clearly defined, valid, reliable, and implemented consistently across all study participants? Q12. Were the outcome assessors blinded to the exposure status of participants? Q13. Was loss to follow-up after baseline 20% or less? Q14. Were key potential confounding variables measured and adjusted statistically for their impact on the relationship between exposure(s) and outcome(s)?
