# Supplementary material for: The current situation and influencing factors of occupational grief among clinical nurses: A scoping review
Source: Medicine (Baltimore). 2025 Dec 19;104(51):e46468. doi: 10.1097/MD.0000000000046468 (PMC12727250; doi:10.1097/MD.0000000000046468)
Supplement: Supplementary file 2 [file medi-104-e46468-s002.docx]

Figure 1. PRISMA 2020 Flow Diagram of the study selection process.

The figure illustrates the identification, screening, eligibility assessment, and final inclusion of studies in the systematic review and meta-analysis. A total of 2,763 records were initially identified from various databases. After removing duplicates, 86 records were screened, resulting in the inclusion of 14 studies.

**Identification of studies via databases and registers**

Records identified from*:

Databases (n = 2763)

PubMed (n= 469), The Cochrane Library (n=0), Wanfang (n=144), Web of Science (n= 892), CINAHL (n=65), PsyclNFO (n= 5), CNKI (n= 19) EMbase (n=1098), CBM (n=20)

Records removed *before screening*:

Duplicate records removed (n = 2677)

**Identification**

Records screened

(n = 86)

Records excluded**

(n = 36)

Reports sought for retrieval

(n = 50)

Reports not retrieved

(n = 0)

**Screening**

Reports assessed for eligibility

(n = 50)

Reports excluded:

Duplicate publication (n=8)

No full text (n=3)

No data (n=2)

Outcome indicators not consistent (n=23)

Studies included in review

(n = 14)

**Included**

*Consider, if feasible to do so, reporting the number of records identified from each database or register searched (rather than the total number across all databases/registers).

**If automation tools were used, indicate how many records were excluded by a human and how many were excluded by automation tools.

Source: Page MJ, et al. BMJ 2021;372:n71. doi: 10.1136/bmj.n71.

This work is licensed under CC BY 4.0. To view a copy of this license, visit <https://creativecommons.org/licenses/by/4.0/>
